# General practitioners knowledge and management of whiplash associated disorders and post-traumatic stress disorder: implications for patient care

**DOI:** 10.1186/s12875-016-0491-2

**Published:** 2016-07-20

**Authors:** Bianca Brijnath, Samantha Bunzli, Ting Xia, Nabita Singh, Peter Schattner, Alex Collie, Michele Sterling, Danielle Mazza

**Affiliations:** Department of General Practice, School of Primary Care, Faculty of Medicine Nursing and Health Sciences, Monash University, Melbourne, Australia; School of Occupational Therapy and Social Work, Curtin University, Perth, WA 6152 Australia; Department of Surgery, St Vincent’s Hospital, The University of Melbourne, Melbourne, Australia; Department of Epidemiology and Preventive Medicine, School of Public Health and Preventive Medicine, Faculty of Medicine Nursing and Health Sciences, Monash University, Melbourne, Australia; Institute for Safety Compensation and Recovery Research, Monash University, Melbourne, Australia; Recover Injury Research Centre, NHMRC CRE in Road Traffic Injury Recovery, Menzies Health Institute, Griffith University, Gold Coast, Australia

**Keywords:** Australia, General practice, Post-Traumatic Stress Disorder, Road traffic crash, Survey, Whiplash

## Abstract

**Background:**

In Australia, general practitioners (GPs) see around two-thirds of people injured in road traffic crashes. Road traffic crash injuries are commonly associated with diverse physical and psychological symptoms that may be difficult to diagnose and manage. Clinical guidelines have been developed to assist in delivering quality, consistent care, however the extent to which GPs knowledge and practice in diagnosing and managing road traffic crash injuries concords with the guidelines is unknown. This study aimed to explore Australian GPs knowledge, attitudes and practices regarding the diagnosis and management of road traffic crash injuries, specifically whiplash associated disorders (WAD) and post-traumatic stress disorder (PTSD).

**Method:**

A cross-sectional survey of 423 GPs across Australia conducted between July and December 2014. We developed a questionnaire to assess their knowledge of WAD and PTSD, confidence in diagnosing and managing WAD and PTSD, frequency of referral to health providers, barriers to referral, and attitudes towards further education and training. Factor analysis, Spearman’s correlation, and multiple ordered logistic regressions were performed.

**Results:**

Overall, GPs have good level knowledge of WAD and PTSD; only 9.6 % (95 % CI: 7.1 %, 12.8 %) and 23.9 % (95 % CI: 20.8 %, 28.2 %) of them were deemed to have lower level knowledge of WAD and PTSD respectively. Key knowledge gaps included imaging indicators for WAD and indicators for psychological referral for PTSD. GPs who were male, with more years of experience, working in the urban area and with higher knowledge level of WAD were more confident in diagnosing and managing WAD. Only GPs PTSD knowledge level predicted confidence in diagnosing and managing PTSD. GPs most commonly referred to physiotherapists and least commonly to vocational rehabilitation providers. Barriers to referral included out-of-pocket costs incurred by patients and long waiting times. Most GPs felt positive towards further education on road traffic crash injury management.

**Conclusion:**

This study has enhanced understanding of the knowledge skills and attitudes of GPs towards road traffic crash injury care in Australia, and has identified areas for further education and training. If delivered, this training has the potential to reduce unnecessary imaging for WAD and optimise the early referral of patients at risk of delayed recovery following a road traffic crash.

## Background

Road traffic crashes (RTCs) place a substantial economic and social burden on Australian society, costing around $27 billion annually [1, 2]. General Practitioners (GPs) see two thirds of people who have experienced a RTC [3] and play a key role in injury diagnosis, monitoring medical complications, managing psychosocial factors that may impede recovery, and assisting in return-to-work assessments [4]. Through appropriate early interventions, GPs can play a crucial role in averting ‘high risk’ patients away from chronicity and disability pathways [5].

However, RTC-related injuries can be complex and challenging to diagnose and manage in general practice due to the lack of clinical markers to predict those at risk of poor recovery and to guide intervention [6]. Whiplash associated disorders (WAD), the most prevalent RTC-related injury [7], are commonly associated with a variety of diverse physical and psychological symptoms that can delay recovery (up to 7 years post injury in some cases) [8]. An estimated one third of individuals with WAD may also have symptoms of Post-Traumatic Stress Disorder (PTSD) [9], and together, pain and PTSD are thought to sustain each other through a complex array of cognitive and behavioural mechanisms that can be difficult to manage in general practice [10]. PTSD may also occur in the absence of physical injury, and because a degree of psychological distress following exposure to a traumatic RTC is common, it can be a challenge for GPs to predict who will go on to experience PTSD and require intervention [11].

Australian clinical guidelines have been developed to assist GPs in the diagnosis of WAD and PTSD, in the identification of patients at risk of chronic pain and disability, and in the selection of referral pathways [11, 12]. The goal of clinical guidelines is to improve the quality and consistency of care across general practice using recommendations based on the best quality of evidence [13]. There is some evidence from elsewhere in the musculoskeletal literature that guideline-adherent care can lead to improved patient outcomes and reduced health-care related costs following injury [14, 15].

However inconsistent uptake of clinical guidelines is an ongoing challenge in general practice [16–19]. Research across diverse areas of clinical practice has identified GP knowledge and attitudes as key barriers or facilitators of guideline uptake [20]. Surveys of GPs in Canada [21] and Scotland [22] have reported negative attitudes towards compensable injury management and limited evidence-based knowledge that may be restricting GP uptake of RTC-related injuries guidelines. Similar findings have been reported by studies in regional Australia. A study in Western Australia examining patients’ and GPs’ experiences of WAD suggested that GPs were not engaging in effective patient-centered care, perceiving themselves to have limited influence on patients’ recovery trajectories [23]. Another Western Australian study found that GPs felt they needed regular training to maintain their confidence levels in treating RTC-related injuries [24]. Similar calls for continued training have been reported amongst Victorian GPs, particularly in the management of psychological injuries following an RTC [25]. Research in rural Queensland showed that insufficient access to specialized rehabilitation services for RTC-related injuries meant that more training was required for GPs to improve care coordination and health service delivery [4]. It remains unknown how generalizable these findings are to the wider population of Australian GPs.

In order to optimise the quality and consistency of RTC-related injury management across general practice in Australia, it is important to understand the facilitators and barriers to guideline uptake on a national scale. Therefore, the aim of this national survey of Australian GPs was to explore GPs’ knowledge, attitudes, and practices regarding the diagnosis and management of RTC-related injuries.

## Methods

### Research design

This observational, cross-sectional study used a questionnaire to survey Australian GPs’ knowledge and attitudes concerning the diagnosis and management of whiplash injury and PTSD.

### Recruitment

From their database of approximately 29,000 GPs, the Australasian Medical Publishing Company provided the research team contact details of 3000 currently practising GPs across Australia. These GPs were randomly selected via a computerised program. Apart from being a currently practising GP, there were no other selection criteria determining study participation. The Australasian Medical Publishing Company database was used because it comprises the most up-to-date, generalizable sample of currently practising GPs from different background (e.g., age, gender, location), and aligns well with the Australian Government Department of Health list of doctors [26].

### Data collection

Following approval by the Monash University Human Research Ethics Committee a package containing an invitation letter, study information sheet, a self-administered questionnaire, and a reply paid envelope was mailed out to 3000 GPs in July 2014. Receipt of the completed survey via the reply-paid envelope implied study consent. A reminder study invitation and survey was sent to 2480 non-responding GPs approximately 2 weeks after the initial mail-out. All questionnaires were labelled with an identification number (ranging from 1 to 3000) to determine the non-responding GPs from the responding GPs. GPs that completed the survey were automatically entered into a draw to win one of three Apple iPad® minis. The data collection period ended in December 2014.

### Instrument development

We could not locate any scales that measured GP knowledge and attitude to RTC-injuries. Therefore we had to develop our own questionnaire through a three-stage process. Stage 1 involved item development through critically reviewing:

- Previous studies on WAD, traumatic injury, and traumatic stress (e.g., [3, 27]),
- Current guidelines for injury diagnosis and management of WAD and PTSD [11, 12], and
- A previous qualitative study on GPs’ management of injured worker return to work [25, 28].

Two authors (BB and NS) led the review of the literature and other members of the team contributed clinical and industry insights (DM and PS are GPs; MS and SB are physiotherapists; AC is an expert on compensable injury). The first draft of the survey included questions on GP demographics, knowledge and attitudes towards WAD and PTSD, patient referral, attitudes to return to work, and areas for further education and training.

Stage 2 involved content validation and item refinement through consultation with four independent experts; two academic-clinicians from general practice and clinical psychology respectively, and two compensable injury industry representatives. These experts were identified based on their experience working with Australian GPs, developing clinical guidelines relevant to WAD and PTSD for RTC-injuries, and/or experience in managing compensable injury claims related to WAD and PTSD.

After these experts supported the content validity of the draft questionnaire, in Stage 3 the survey was pilot tested over the phone with a total of 15 GPs to confirm content reliability and face validity. These 15 GPs had participated in the research team’s previous qualitative study on GPs’ management of injured worker return to work following compensable injury [25, 28]. They had recent experience (<12 months) treating compensable injury patients injured either at work or in transport accidents and had previously provided consent to be contacted about future research. GPs that had participated in the pilot survey were excluded from the final study. Stages 2 and 3 resulted in refinements to improve item clarity and wording rather than content revisions.

The final questionnaire consisted of four categories of information:

- ***Demographic characteristics:*** Data were collected regarding the respondent’s age, gender, number of years’ experience in general practice, location of practice, state where practice is based, number of GPs in the same practice, and types of injuries observed within the last 3 years.
- ***Knowledge about WAD and PTSD and confidence in injury diagnosis and management:*** Knowledge about WAD and PTSD was measured with 12 and 15 statements respectively. Response choices as “True”, “False” and “Don’t know” were provided. Prior to analysis, for each statement a score of “1” was given for correct response while incorrect and don’t know responses were given a score of “0”. To assess the respondents’ level of accurate knowledge, new variables labelled “total knowledge score of WAD/PTSD” were then created by summing their knowledge scores. The mean and standard deviation of knowledge scores were used to categorise GPs into three groups: poor, moderate, and high knowledge.
  GPs were also asked to rate their level of agreement to statements relating to confidence of injury diagnosis and management using a five-point Likert scale (strongly disagree to strongly agree, scored from “1” to “5”). This was then reduced to the three levels by combining all “disagree” and “agree” responses into categories of “lower” and “higher”, “not sure” responses were treated as “moderate”.
- ***Patient referral:*** The third section of the questionnaire explored GPs’ frequency of patient referral to health providers, and barriers to patient referral to health providers. These questions were also measured using a five-point Likert scale (from “strongly disagree to “strongly agree”).
- ***Patient referral and further education and training:*** The fourth section focused on exploring GPs’ attitudes towards further education and training needs on RTC injury management. Six potential areas of education were suggested and perceived need was measured by a five-point Likert scale.

### Statistical analysis

First, descriptive analyses including frequencies and percentages of demographic information, and knowledge and attitudes statements were conducted. Chi-square tests were performed to compare knowledge level regarding WAD and PTSD among different demographic groups. Second, ordered logistic regression was performed to examine predictors of GPs’ confidence in diagnosing and managing WAD and PTSD. The variable was coded as “1” representing “lower confidence”, with “moderate confidence” and “higher confidence” coded as “2” and “3” respectively. Predictor variables included demographic characteristics and knowledge variables. Since the variable “years of experience” is highly correlated with “age”, the variable “age” was excluded from the model. Multivariable ordered logistic regression analysis was performed to identify the associated factors. Third, an explorative factor analysis (principal components analysis, varimax rotation) was conducted among variables of barriers related to GPs’ decision to refer RTC injury patients to allied health providers. The new variables generated by factor analysis were further entered in to a Spearman’s correlation analysis to explore GPs’ perceived barriers with their referring rate of allied health providers. A Spearman’s correlation analysis was also conducted to explore GPs’ confidence of diagnosing and managing WAD/PTSD and their attitudes towards further relevent education and training.

## Results

### Participant demographics

In total, 423 GPs returned completed questionnaires, yielding a response rate of 13.9 %. Table 1 shows the demographic profile of participating GPs and the most common injuries presenting to their practice in the last 3 years. The most common RTC injuries seen were muscular or soft tissue bruises and sprains (96 %), followed by injuries to the neck (83 %) and psychological conditions such as PTSD (57 %). GPs reported that less than a quarter (21 %) had no injuries at all and about half (49 %) were cases requiring ongoing management after initial presentations to hospitals.

**Table 1:** GPs’ demographic profile and their most commonly seen RTC-related injuries

Demographic	n	(%)	Most common injuries presenting to GPs’	n	(%)
Age			Injury type		
25–35 years	50	12	No injury at all	88	21
36–45 years	96	23	Muscular or soft tissue bruise or strain	406	96
46–60 year	168	40	Whiplash injury to the neck	351	83
60+ yrs	109	26	Back injury - thoracic	199	47
Gender			- lumbar	168	40
Male	214	51	Patients with one or more fractures	205	48
Female	209	49	Head injury	152	36
Years of experience as a GP^a^			Concussion	159	38
≤ 10 year	102	24	Psychological conditions such as PTSD	243	57
11–20 year	50	12	Skin wounds	196	46
20+ yrs	270	64	Injuries requiring ongoing management post-hospital discharge	207	49
Location of practice			Joint injury - upper limb	134	32
Urban	251	59	- lower limb	112	26
Regional	139	33	Chronic pain	240	57
Remote	33	8			
Overall no. of GPs in practice^b^					
1	41	10			
2–3	71	17			
4–7	169	40			
≥ 8	139	33			
States					
VIC	106	25.1			
NSW	126	30.5			
QLD	92	21.7			
SA/NT/WA/TAS/ACT	99	22.7			

^a^ *n* = 422 (1 missing) ^b^ *n* = 420 (3 missing)

### Knowledge of WAD and PTSD

Table 2 shows the percentage of correct responses for items examining GPs’ knowledge of WAD injury and PTSD. These ranged from 20 % to 95 % (mean = 73, SD = 25.96) for WAD and from 22 % to 96 % (mean = 68. SD = 23.45) for PTSD. In general, the majority of GPs were able to correctly identify indicators of a poorer prognosis for patients with WAD, but there were gaps in GPs’ knowledge about pain intensity being a prognostic indicator for poor recovery (Table 2, item 1-a, 38 % correct). Furthermore 44 % of GPs correctly identified that an inability to rotate the neck beyond 45° to the left or right was an indicator for cervical spine x-ray (Table 2, item 2-a). GPs also lacked knowledge on appropriate treatment for WAD with 80 % of GPs incorrectly identifying manipulation (Table 2, item 3-b) as a first line evidence-based treatment.

**Table 2:** Responses for items dealing with knowledge about WAD and PTSD

Item	T/F	% correct
Whiplash		
1. People with a whiplash injury to the neck have a poorer prognosis when:		
a. Pain > 7/10 on a Visual Analogue Scale on day 1 vs follow up	T	38
b. People have a psychological injury or psychological comorbidity	T	95
c. People have a low expectation of recovery	T	90
d. People do not have an expectation of return to work	T	87
2. Components of a whiplash injury to the neck that indicate the need for cervical spine X-ray include:		
a. Inability to rotate the neck beyond 45° to the left or right	T	44
b. Paraesthesia in the extremities	T	95
c. Age ≥ to 65 years	T	65
3. Effective evidence-based treatments for whiplash injury to the neck include:		
a. Range of movement exercises	T	95
b. Manipulation	F	20
c. Passive mobilisation	F	73
d. Collars	F	76
e. Rest in bed rather than graded return to usual activities	F	96
Average		73
PTSD		
4. People who are likely to have a poorer prognosis from PTSD if untreated include:		
a. People who have PTSD symptoms persisting beyond 1 month after the injury	T	85
b. People whose physical injuries are of greater severity	F	34
c. People involved in litigation for criminal or civil purposes	T	84
5. I would usually refer a patient with a post-traumatic stress presentation to mental health services if there was:		
a. Prominence of depression in the presentation	T	81
b. Prominence of pain in the presentation	T	31
c. Presence of PTSD symptoms subsequent to a mild traumatic brain injury	T	72
d. Presence of PTSD symptoms persisting beyond 1 month post injury	T	82
e. Presence of a mental health history	T	76
6. First line evidence-based treatments for adults with PTSD include:		
a. Trauma focused cognitive behaviour therapy (CBT)	T	89
b. Selective Serotonin Reuptake Inhibitors (SSRI)	F	22
c. Benzodiazepines	F	85
7. Aside from PTSD, psychiatric disorders that can commonly occur following a injury include:		
a. Major Depressive Disorder	T	83
b. Generalized Anxiety Disorder	T	94
c. Brief Psychotic Disorder	F	49
d. Substance Use Disorder	T	59
Average		68

For questions relating to PTSD diagnosis, 31 % of GPs correctly responded that they would usually refer a patient with a PTSD presentation to mental health services if there was prominence of pain in the presentation (Table 2, item 5-b) and 34 % (186/420) of GPs correctly identified physical injuries of greater severity as not an indicator of poorer prognosis for PTSD (Table 2, item 4-b). Selective serotonin reuptake inhibitors (SSRIs) were identified by 22 % of GPs as not being a first line evidence based treatment for adults with PTSD (Table 2, item 6-b).

Overall, 32.2 % (95 % CI: 27.9 %, 36.9 %) of GPs were deemed to have a high knowledge of WAD, whilst 58.2 % (95 % CI: 53.4 %, 62.9 %) and 9.6 % (95 % CI: 7.1 %, 12.8 %) of GPs were deemed to have moderate and low knowledge of WAD respectively. The majority of GPs (59.4 %, 95 % CI: 54.6 %, 64.0 %) had moderate knowledge of PTSD, and 16.7 % (95 % CI: 13.4 %, 20.6 %) had low and 23.9 % (95 % CI: 20.0 %, 28.2 %) had a high knowledge of PTSD. In addition, significant associations were found between level of WAD knowledge and GPs’ age (*p* < 0.001), location of practice (*p* = 0.020), and the overall number of GPs in a practice (*p* = 0.009). Location of practice was also found to be associated with GPs’ level of knowledge in PTSD (*p* = 0.001).

### Factors associated with confidence in diagnosing and managing WAD and PTSD

Table 3 shows the results of the multivariate ordered logistic regression analyses for the factors associated with GPs’ confidence in diagnosing and managing WAD and PTSD. The results suggest that female GPs were less confident in diagnosing WAD than their male counterparts (OR = 0.54, 95 % CI, 0.31–0.96). GPs with more than 20 years’ experience were more confident in diagnosing and managing WAD compared to those with ≤10 years’ experience. Meanwhile, GPs from urban locations were 2.45 times (95 % CI, 1.04–5.80) more confident in diagnosing WAD than remote GPs, and regional GPs were more confident in managing WAD than remote GPs. GPs’ knowledge of WAD was a significant predictor of confidence in diagnosing and managing WAD.

**Table 3:** Factors associated with levels of confidence in diagnosing and managing WAD and PTSD

	Diagnosing WAD	Managing WAD	Diagnosing PTSD	Managing PTSD
	OR (95 % CI)	OR (95 % CI)	OR (95 % CI)	OR (95 % CI)
Gender				
Male	1 (ref^a^)	1 (ref)	1 (ref)	1 (ref)
Female	0.54 (0.31–0.96)^*^	0.72 (0.46–1.14)	1.08 (0.67–1.66)	0.99 (0.68–1.47)
Years of experience as a GP				
< or equal to 10 year	1 (ref)	1 (ref)	1 (ref)	1 (ref)
11–20 year	2.19 (0.89–5.34)	2.38 (1.09–5.18)^*^	1.17 (0.60–2.43)	0.73 (0.38–1.41)
20+ yrs	2.78 (1.50–5.17)^*^	2.26 (1.34–3.81)^*^	1.61 (0.95–2.73)	1.04 (0.65–1.68)
Location of practice				
Remote	1 (ref)	1 (ref)	1 (ref)	1 (ref)
Regional	1.90 (0.77–4.61)	2.91 (1.29–6.55)^*^	0.66 (0.27–1.62)	0.54 (0.24–1.23)
Urban	2.45 (1.04–5.80)^*^	2.03 (0.93–4.39)	0.50 (0.21–1.21)	0.53 (0.24–1.18)
Overall no. of GPs in practice				
1	1 (ref)	1 (ref)	1 (ref)	1 (ref)
2–3	2.46 (0.88–6.87)	2.18 (0.91–5.19)	0.96 (0.43–2.13)	1.11 (0.53–2.33)
4–7	1.82 (0.78–4.27)	1.38 (0.66–2.89)	1.54 (0.74–3.18)	0.94 (0.49–1.83)
> or equal to 8	1.42 (0.59–3.45)	1.28 (0.59–2.76)	1.06 (0.51–2.19)	1.00 (0.51–1.98)
WAD knowledge level				
Low	1 (ref)	1 (ref)		
Middle	2.38 (1.05–5.39)^*^	2.13 (1.05–4.30)^*^	-	-
High	2.86 (1.15–7.12)^*^	2.93 (1.36–6.32)^*^		
PTSD knowledge level				
Low			1 (ref)	1 (ref)
Middle	-	-	1.85 (1.08–3.16)^*^	1.73 (1.04–2.88)^*^
High			3.27 (1.68–6.37)^*^	2.42 (1.34–4.360^*^

^a^Reference group

^*^Significant at *p*-value <0.05

For diagnosing and managing WAD and PTSD, only GPs’ PTSD knowledge level was found to predict confidence in diagnosing and managing PTSD, but gender, years of experience, location of practice, and number of GPs in practice were not found to be predictors (Table 3).

### Patient referral

GPs most commonly referred their patients to physiotherapists (94 %), followed by orthopaedic specialists (53.4 %) and mental health professionals (51.6 %). Occupational physicians and vocational rehabilitation providers were referred to least by GPs (30.7 and 30.4 %, respectively).

The factor analysis identified two factors that could impact GPs’ decisions to refer injury patients to allied health providers (Table 4). Factor I could be interpreted as barriers relating to financial and time concerns (‘money and time’), while factor II described uncertainties about the role that allied health providers could play in injury management (‘uncertainties’). As shown in Table 5, concerns about money and time were positively correlated with likelihood of referring to physiotherapists (*r* = 0.20, *p < 0.001*), mental health professionals (*r* = 0.21, *p < 0.001*), occupational physicians (*r* = 0.12, *p = 0.02*) and vocational rehabilitation providers (*r* = 0.13, *p = 0.01*). Negative associations were found between “uncertainties about allied health providers” and physiotherapist referral (*r* = −0.17, *p < 0.001*), and between vocational rehabilitation provider referral (*r* = −0.16, *p = 0.002*).

**Table 4:** Factor analysis of barriers impacting GPs’ decisions to refer patients to allied health providers (principal component analysis, varimax rotation)

	Response on 5-point Likert scale					Factor analysis	
	Strongly disagree (%)	Disagree (%)	Not sure (%)	Agree (%)	Strongly agree (%)	Factor I	Factor II
Out of pocket costs incurred by patients	1.7	25.5	4.5	44.5	23.8	0.92	
Long hospital/community waiting lists	1.7	27.3	5.9	39.3	25.8	0.92	
Uncertainty of clinical benefit	4	40.5	16.1	34.4	5		0.75
Uncertainty of allied health professional’s role	6.2	61.4	11.4	17.9	3.1		0.84
Uncertainty of role played by vocational rehabilitation providers	3.6	43.5	16.9	29.5	6.7		0.81
Uncertainty in coordinating allied health injury management services	3.4	42.8	17.8	31.5	4.4		0.79

**Table 5:** Correlations (Spearman’s) between factors of barriers to referrals and referred health providers

	Response on 5-point Likert scale					Spearman’s correlation	
	Strongly disagree (%)	Disagree (%)	Not sure (%)	Agree (%)	Strongly agree (%)	Factor I	Factor II
Physiotherapists	44.4	49.9	1.2	4	0.5	0.20^*^	−0.18^*^
Mental health professionals	6.9	44.7	16.9	30.9	0.7	0.21^*^	0.00
Occupational physicians	5.1	25.6	19.3	45.4	4.6	0.12^*^	−0.10
Orthopaedic specialists	7.9	45.5	12.7	33	1.0	0.09	−0.02
Vocational rehabilitation providers	4	26.4	26.9	38.8	4	0.13^*^	−0.16^*^

^*^Significant at *p*-value <0.05

### GP education and training needs

The majority of GPs felt positive towards further education about the diagnosis and management of psychological conditions following an RTC (Table 6). A positive attitude was negatively associated with GPs’ confidence in diagnosing and managing WAD (*r* = −0.20, *p* < 0.001). A similar association was found between positive attitudes towards further education on guidelines for acute WAD and GPs’ confidence in diagnosing and managing WAD (*r* = −0.16, *p* < 0.001). Furthermore, most GPs had a positive attitude towards further education about diagnosis and management of psychological conditions following a RTC, but no significant association was found between positive attitude and GPs’ confidence in diagnosing and managing PTSD.

**Table 6:** Correlations (Spearman’s) between confidence of diagnosing and managing WAD/PTSD and futher education and training

	Response on 5-point Likert scale					Spearman’s correlation	
	Strongly disagree (%)	Disagree (%)	Not sure (%)	Agree (%)	Strongly agree (%)	Diagnosing and managing WAD	Diagnosing and managing PTSD
Diagnosis and management of whiplash injury to the neck following a road traffic crash	0	2.6	6.4	62.3	28.7	−0.20*	
The guidelines available for acute whiplash management	0.2	1.4	6.2	60.2	32	--0.16*	
Diagnosis and management of psychological conditions following a road traffic crash	0	2.4	5.9	62.6	29.1		−0.09

^*^Significant at *p*-value <0.05

## Discussion

Amongst the responders to this national survey of Australian GPs, overall knowledge regarding the evidence-based diagnosis, management and prevention of RTC-related injuries may be considered to be moderate to high. Approximately 10 % and 17 % of GPs who responded were considered to have low evidenced-based knowledge for WAD and PTSD respectively. The level of knowledge reported by the GPs in this study also appears to be relatively higher than that reported in previous studies conducted in international contexts [21, 22]. A survey of Canadian GPs identified negative attitudes towards the management of patients with WAD with a limited understanding of diagnostic indicators [21]. A survey of Scottish GPs identified a knowledge gap as the reason for poor recognition and management of PTSD in general practice [22]. As the present study had a significantly lower response rate compared to the Canadian and Scottish studies, it is possible that our sample represents a subgroup of GPs with a higher knowledge of RTC-related injuries than their peers. Alternatively, given that nearly 40 % of our sample were from regional and remote areas, it is possible that limited access to specialised rehabilitation services compelled GPs to more frequently manage RTC injuries on their own and thus their confidence may be related to the frequency of managing such cases. Previous studies documenting the perspectives of rural GPs from Western Australia and Queensland suggest this is the case at least regarding WAD [23, 24]. GP knowledge about PTSD is more difficult to benchmark since the only other Australian study exploring this topic – a qualitative investigation in urban Melbourne, Victoria – did not specifically examine PTSD but the entire spectrum of mental conditions related to compensation claims management [25].

The findings of this study also highlight important gaps in GPs’ knowledge. Only 44 % of GPs who responded correctly identified that an inability to rotate the neck beyond 45° to the left or right was an indicator for cervical spine x-ray after neck injury. A lack of awareness of when to refer to x-ray may help to explain the relatively high rates of referral (34 % of all WAD claims) for imaging investigations that have been reported following neck injuries in Australia [7]. Routine referral for imaging following neck injury is not recommended as imaging findings are not associated with pain and disability following spinal injury [29, 30]. The presence of so-called ‘abnormalities’ seen on radiological investigation may be incidental findings that are prevalent amongst the asymptomatic population, and can function as ‘red-herrings’ in the management of the patients’ symptoms [31]. To reduce the impact of such ‘red-herrings’ on patient management and the costs associated with unnecessary imaging, a national strategy may be required to raise awareness amongst Australian GPs of evidence-based indicators for imaging referral following neck injury.

It is interesting to note that only 20 % of GPs correctly answered that manipulation is *not* an effective evidence-based treatment for WAD. The endorsement of manipulation by 80 % of the GPs in the present study contrasts with findings from a Canadian survey involving 362 GPs which reported that only 16 % of GPs believed manipulation to be useful [21]. However it is comparable with findings from a survey in the Australian state of Queensland that found 84 % of physiotherapists agreed with the statement that manipulation/mobilisation is effective in the management of most patients with acute whiplash injury [32]. To encourage consistencies in the evidence-based treatment of WAD across Australia, further clinical training in this area might be helpful.

Similarly, to ensure consistencies in the management of PTSD across Australia, further clinical training may be needed to educate GPS about the potential benefits of early psychological referral in patients presenting with PTSD and pain [33, 34]. Evidence-based guidelines for PTSD management recommend trauma focused cognitive behavioural therapy (CBT) as the first-line treatment for PTSD following a RTC and recommend against the routine use of pharmacotherapy to treat PTSD except when a patient’s symptoms cannot be managed by psychological means alone [11]. Whilst 89 % of the GPs who responded agreed with the statement that CBT is a first-line treatment for PTSD, 58 % also agreed that SSRIs are a first-line evidence based treatment. This may imply that the GPs surveyed endorse the combination of psychological and pharmacotherapy in the management of PTSD following a RTC despite the lack of evidence regarding the potential benefits of combining psychological and pharmacotherapy [11, 35]. Further, amongst the GPs surveyed there appeared to be a low awareness of the impact of pain on PTSD symptoms with only 31 % of GPs agreeing that they would usually refer a patient with a post-traumatic stress presentation to mental health services if there was prominence of pain in the presentation and only 34 % agreeing that physical injuries of greater severity are not an indicator of poorer prognosis for PTSD. It may be important to raise awareness amongst Australian GPs of evidence suggesting that symptoms of pain can drive and compound PTSD following an RTC (and vice-versa), increasing the risk of chronicity and disability [5, 27, 36].

Cost, time, and uncertainty were all endorsed as barriers to referral following RTC-related injuries. Whilst high out of pocket costs and long waiting lists for secondary care are well-documented in Australia [37, 38], widespread uncertainty of the role of the health provider as a barrier to RTC-related injury referral has not been described. Ensuring that GPs understand not only who they can refer their patients to but why, may help facilitate access to appropriate early interventions.

In this study, a responder’s knowledge of WAD and PTSD was a significant predictor of confidence in diagnosing and managing WAD and PTSD. Greater years of experience as a GP (>20 years), male gender, and urban practice location were all independently associated with greater self-reported confidence. This is consistent with surveys of GPs’ confidence in diagnosis and management in other areas of clinical practice [39, 40]. Providing training opportunities for those GPs that are less confident in their ability to diagnose and manage WAD and PTSD may be important as clinician confidence is likely to influence adherence to guidelines in clinical practice [41]. Cross sectional and qualitative data suggest that low GP confidence in integrating patient preferences with guideline care presents a key barrier to guideline adherence [42, 43]. For example, a Dutch study involving interviews with 20 GPs and 20 of their patients with spinal pain found that GPs would depart from guideline best practice and refer to imaging if their patients requested it and they could not convince them that it was not indicated [44]. Up-skilling GPs to optimise their communication in the clinical encounter with patients presenting with ‘complex’ RTC-related conditions such as WAD and PTSD may improve GP confidence and adherence to guideline care.

Of interest in this study, most responders reported that they were likely or very likely to attend further education and training sessions on the management of RTC-related injuries, regardless of their level of knowledge or confidence. Responders from urban, rural, and remote settings, and responders from across all states of Australia were equally likely to endorse further education. Despite this self-reported interest, engaging time-poor GPs in training and education in RTC-related injuries can be challenging. Indeed, an online education program to disseminate the Australian WAD guidelines in the state of New South Wales was found to be effective in improving knowledge of WAD guidelines, including when to refer for cervical x-rays and what interventions to provide patients; however, the uptake of the program was relatively low [3]. Low uptake of online education by Australian GPs may be a common trend, despite being an accessible, cost and time-effective education medium [45, 46]. Research is needed to better understand and overcome the barriers to GP engagement in online education and understand GPs’ preferences for education on RTC-related injuries in the future.

### Strengths and limitations of the study

This is the first national survey in Australia to explore GPs’ knowledge, attitudes, and practices regarding the diagnosis and management of RTC-related injuries. Our sample represents GPs from a range of sociodemographic and geographical backgrounds. This study has generated some important findings, highlighting key gaps in GPs’ knowledge that can be addressed to optimise the uptake of RTC-related injury guidelines in Australia.

However, several limitations in this study should be noted. First, the questionnaires were self-completed and all the analysis was based on self-reported data. Therefore response bias may explain in part why high proportions of respondents selected positive options (e.g., I am very likely to attend further training) for many items. Second, the response rate for this study was low, although not unprecedented in surveys of the GP population, known as a hard-to-reach target group [47]. Responders may represent a subgroup of GPs who differ in some way from the remaining population, such as time availability, percentage case-load of RTC-related injuries or special interest in RTC-related injuries. Unfortunately, the GP dataset provided by the Australian Medical Publishing Company to the research team did not include demographic details about the GPs, hence it is difficult to ascertain how study participants differed from non-responders. However, it has been proposed that in surveys involving physicians this may be less of a threat due to the reasonably homogenous structure of the study population, i.e., GPs, in terms of professional knowledge, education, behaviour, and attitudes [48, 49].

Responders’ special interest in RTC-related injuries may also in part explain the relatively high knowledge of evidence based practice in the diagnosis and management of WAD and PTSD. It remains to be seen if this knowledge is reflected in adherence to guidelines in clinical practice. Indeed, an Australian study found that GPs with self-declared ‘special interest’ in low back pain appeared to be less likely to adhere to low back pain guidelines in clinical practice than GPs without a ‘special interest’ [50]. As GPs with a special interest had a higher case load of patients with low back pain than GPs without a special interest, Buchbinder et al. [50] proposed vested financial and/or professional interests as one explanation for non-adherence to guidelines in clinical practice. Further research is required to determine whether GPs who are more confident and have a higher level of evidence based knowledge are more or less likely to adhere to clinical guidelines in the diagnosis and management of WAD and PTSD.

## Conclusions

In exploring GP knowledge and attitudes about the diagnosis and management of RTC-related injuries we have highlighted the potential to reduce unnecessary imaging following WAD and the potential to optimise the early referral of patients at risk of delayed recovery following a RTC injury. As GP self-reported uptake of guidelines may be distinct from adherence to guidelines in clinical practice, we call for future research using alternative methodologies such as analysis of GPs’ records. Building this detailed picture of RTC-related injury care in Australian general practice will help bring us towards our objective of ensuring that all Australians injured in an RTC have access to high quality and consistent care.

## Abbreviations

CI, Confidence interval; GP, General practitioner; OR, Odds Ratio; PTSD, Post traumatic stress disorder; RTC, Road traffic crash; SD, Standard deviation; WAD, Whiplash Associated Disorder.
